# Elektra complex in dissociative identity disorder: A case report

**DOI:** 10.1192/j.eurpsy.2021.1815

**Published:** 2021-08-13

**Authors:** P. Jain, V. Mudgal, U. Sardesai, V. Pal

**Affiliations:** Psychiatry, MGM Medical College, INDORE, India

**Keywords:** Dissociative Identity Disorder, psychotherapy, cultural psychiatry, Elektra Complex

## Abstract

**Introduction:**

Dissociative identity disorder is a quite popular psychiatric diagnosis in general public but in actuality has a very low prevalence rate. Dissociative identity disorders are characterized by disruption of identity characterized by two or more distinct personality states with its own memories, behaviour, and preferences.

**Objectives:**

Authors present a case report about a patient of Dissociative identity disorder with Elektra complex as unconscious conflict.

**Methods:**

A case report along with literature review forms the basis of discussion.

**Results:**

Mrs A, 30 years female, a housewife, belonging to low socioeconomic status, reported to the OPD, along with her husband. About 1 year ago her family members noticed that her behaviour and action became altered. Such alteration in behaviour was only episodic. A detailed evaluation was done and a diagnosis of Dissociative identity disorder was established. The treatment included psychotherapy facilitated by hypnosis addressing the conflict along with escitalopram 10 mg once a day and clonazepam 0.5mg at night, clonazepam was tapered and stopped within 1 month while escitalopram was hiked upto 20 mg and patient improved along with decrease DES scores.

**Conclusions:**

In Freudian psychology the girl child identifies with her mother and represses her sexual feelings toward her father commonly known as the Elektra complex. In spite of trance and possession syndrome being more prevalent in countries like India, we urge to keep dissociative identity disorder as a differential in order to catch the eye of the clinicians and researchers on the recognition of clinical manifestation and exploration of therapeutic strategies.

**Disclosure:**

No significant relationships.

